# Understanding the Effects of Smart-Speaker-Based Surveys on Panelist Experience in Immersive Consumer Testing

**DOI:** 10.3390/foods12132537

**Published:** 2023-06-29

**Authors:** Ashley M. Soldavini, Hamza Diaz, John M. Ennis, Christopher T. Simons

**Affiliations:** 1 Department of Food Science & Technology, The Ohio State University, 2015 Fyffe Rd., Columbus, OH 43210, USA; soldavini.1@osu.edu; 2 Aigora LLC, 2515 Whispering Oaks Ct., Midlothian, VA 23112, USA; hamza.diaz@aigora.com (H.D.); john.m.ennis@aigora.com (J.M.E.)

**Keywords:** engagement, System Usability Scale score, Amazon Alexa, sensory testing, immersive technologies

## Abstract

Utilizing immersive technologies to reintroduce the environmental context (i.e., visual, auditory, and olfactory cues) in sensory testing has been one area of research for improving panelist engagement. The current study sought to understand whether pairing smart-speaker questionnaires in immersive spaces could positively affect the panelist experience through enhanced ecological validity. To this end, subjects performed an immersive consumer test in which responses were collected using a traditional computer-based survey, a smart-speaker approach incorporating a direct translation of the computer questionnaire into a verbal survey requiring numeric responses, and an optimized smart-speaker survey with alternative question formatting requiring spoken word-based responses. After testing, participants answered the Engagement Questionnaire (EQ) to assess participant engagement during the test, and the System Usability Scale (SUS) survey to understand the ease, and potential adoption, of using the various survey technologies in the study. Results indicated that the traditional computer-based survey was the most engaging (*p* < 0.001) and usable (*p* < 0.001), with no differences found between the two smart-speaker surveys (*p* = 0.803 and *p* = 0.577, respectively). This suggests that the proposed optimizations for the smart-speaker surveys were not robust enough to influence engagement and usability, and further research is needed to enhance their conversational capabilities.

## 1. Introduction

The complexity of designing sensory and consumer studies lies in balancing experimental control with a realistic consumer experience. In-hall testing is typically performed in a sterile environment with participants sitting in individual booths to prevent them from interacting with one another [1]. While this allows for the greatest level of experimental control and inhibits various sources of bias, it fails to capture a real-life experience in which interactions with the outside world might influence perception and liking [2,3]. To account for the relevant context during testing, an in-home study design can instead be used. In this scenario, participants use and evaluate a product in their everyday lives, capturing the realistic consumer experience [4]. However, even with the clearest test instructions, it is impossible to guarantee that all participants complete the evaluation correctly, or even try the stimuli in question. This suggests the ideal test scenario is a middle ground of these two opposing test designs, one that allows for not only experimental control but also the relevant environmental context. 

Previous immersive studies have shown that it is possible to re-introduce the relevant environmental context to in-hall testing, resulting in increased product discrimination and panelist reliability [5,6]. To be effective, special consideration needs to be given to selecting appropriate environmental cues for the set of stimuli that are generic enough for all participants to relate to [7]. Even if the appropriate environmental context is found, the study may not be fully ecologically valid, as the device used for data collection (i.e., tablet) is not commonly used to express opinions in a real-life scenario. Typically, a person would verbalize their thoughts towards a certain product to an accompanying friend or family member [8]. For this reason, using devices with voice-assisted technology to administer surveys in immersive testing may further enhance a participant’s experience. One such device that could be used is the smart speaker, a voice-assisted technology with an integrated personal assistant that interacts with users verbally to perform tasks, such as setting reminders, playing music, and answering questions [9].

Although a relatively new technology, the use of smart speakers in e-commerce has been assessed [10,11,12]. The features that encourage consumers to utilize these devices have been identified and include the perceived ease of use, perceived usefulness, and quality of the system [10]; however, concerns with privacy impede user adoption [11]. Moreover, consumer engagement and behavioral responses have been shown to be positively enhanced via the use of high-quality, interactive artificial intelligence devices [12]. However, to date, no study has specifically embedded smart-speaker technology into the workflow associated with sensory and consumer product testing.

Before implementing a new survey technology, it is important to understand how participants feel about the new experience. Two metrics to capture this information are panelist engagement and system usability. Participant engagement has been shown to be a key metric associated with panelist reliability and performance [2]. While engagement is a multi-dimensional construct that can be measured in a variety of ways depending on the field of research, engagement in sensory and consumer testing focuses on three main factors (Affective Value, Purposeful Intent, and Active Involvement) that encompass the panelist experience (interest, personal relevance, and focus and attention, respectively) [13]. Prior research suggests that a participant who is more engaged in a task generally provides more consistent feedback over time and has improved discrimination between stimuli [5]. System usability provides a measure for how easy or difficult a new technology is to learn and use [14,15]. Prior meta-analyses of SUS results performed by Lewis and Sauro revealed that an SUS score of 68 is the minimum required score to label a technology as usable [15,16]. A device that receives a higher System Usability Scale (SUS) score is easier to use and can be adopted by the general population without training. Therefore, a new survey technology should only be deployed if it receives high ratings for both panelist engagement and system usability. However, currently, there are no studies investigating the effect of smart-speaker technology in sensory and consumer testing on panelist engagement and usability.

This study aims to address this knowledge gap by investigating the effect of smart speakers and the questionnaire style on the user experience (i.e., engagement and usability) in an immersive consumer test environment. A computer-based survey, numeric-based smart-speaker survey, and word-based smart-speaker survey were compared. It was hypothesized that the smart-speaker surveys would be more engaging in the immersive test environment. Additionally, it was thought that optimizations made to the word-based smart-speaker survey would further increase engagement by making the test experience more conversational. With respect to usability, it was hypothesized that the computer-based survey would receive a higher system usability rating compared to the smart-speaker format, as participants were most familiar with this system. Lastly, it was expected that the smart-speaker surveys would score above the defined benchmark for a usable technology, supporting their use in sensory and consumer testing.

## 2. Materials and Methods

### 2.1. Subjects 

Participants were recruited from Ohio State University (OSU) Department of Food Science’s Consumer Sensory Database using an online screening questionnaire. This screener was created using Qualtrics XM web-based survey development software (Provo, UT, USA). To qualify for the study, respondents had to select that they consumed the three categories of dips being evaluated (i.e., salsa, guacamole, and queso), as well as use Amazon Alexa-powered smart speakers, as this was the platform being used in the study. Individuals that selected that they had food allergies or sensitivities, or were currently suffering from ailments that impacted their ability to smell, taste, or hear were excluded from the study. Participants also needed to select that they were willing to attend all required test sessions (once per week for three consecutive weeks). Those that met all the criteria for the study were invited to participate and registered for test sessions on a first-come first-served basis.

To determine the minimum number of participants required for the study, a power analysis was performed using an effect size of a 1-point difference in the Engagement Questionnaire (EQ) and a midrange standard deviation (σ = 1.25, α = 0.05, power = 80%) based on data from previous studies using the EQ [17]. The results indicated a sample size of 30 subjects. However, to be consistent with previous studies assessing hedonic consumer testing and its relation to engagement, as well as increase the power and likelihood of finding differences in later analyses outside the scope of this study, a larger sample size was selected. The final number of participants used was 58, with 57 successfully completing all three test sessions. Data collected from the participant that left the study was discarded. Of the 57 participants, 20 were male and 37 were female, and they ranged in age from 18 to 75 years old. Each panelist was compensated for their participation in the study, receiving USD 15 after their first two sessions and USD 30 after the final session. The increase in compensation for the last session is a common technique used for maintaining panelist attendance in multi-day studies.

### 2.2. Ethics

The research protocol for this study was submitted to and approved by OSU’s Institutional Review Board. This approved protocol (IRB-2020B0312) included details such as consent procedures, approved communications to participants, recruitment procedures, experimental procedures, and compensation. All participants were required to sign an Informed Consent form before being allowed to test and were provided the opportunity to ask any questions at this time. The compensation structure was also discussed before testing commenced.

### 2.3. Materials

#### 2.3.1. Stimuli 

Test samples consisted of three commercial salsas (both refrigerated and shelf-stable), three guacamoles (refrigerated), and three quesos (shelf-stable). These were purchased on a weekly basis to ensure product freshness, which was especially important for the refrigerated products (i.e., guacamoles and fresh salsas). To vary samples within a dip category, Mesquite Liquid Smoke (The Colgin Companies, Dallas, TX, USA) was added at various levels. Table 1 displays the specific dips (including brands) and the amount of liquid smoke added to each sample. Smoke addition levels were determined through benchtop tastings using 2–3 members of the Simons Research Group, ensuring they were differentiable and approximately equal in intensity across product types. The resulting sample sets consisted of a zero-smoke sample, a low-intensity smoke sample, and a high-intensity smoke sample for each of the tested dip categories. Product preparation occurred the afternoon before testing to allow the flavors to meld together and sit overnight in a refrigerator (held at 6 °C), following a product preparation protocol (see Appendix A). On the morning of test day, samples were portioned into 3.25 oz (i.e., 96 mL) translucent plastic souffle cups (Choice Brand, WebstaurantStore Food Service Equipment and Supply Company, Lancaster, PA, USA), pre-labeled with numeric 3-digit random codes that corresponded to pre-labeled 4 oz (i.e., 118 mL) molcajetes (Model NHS1008, H.S. Inc., Oklahoma City, OK, USA) used for serving. Products were served in the same style molcajetes that participants saw in the restaurant video recording for congruency. These pre-portioned samples were placed back in the refrigerator until testing. 

#### 2.3.2. Questionnaire Devices

Three product questionnaires were utilized in this study, and each were executed using a different electronic device. Participants used an iPad Air tablet (Apple Inc., Cupertino, CA, USA) for data collection in the Control condition. Third-generation Amazon Echo Dots (Amazon.com, Inc., Seattle, WA, USA) were used for the test surveys (i.e., Translated and Optimized). Each smart-speaker survey had its own dedicated Echo Dot to ensure the appropriate questionnaire was being administered from the experimental design. 

After each product questionnaire, the EQ and SUS were completed on the iPad Air tablet in a separate non-product-related survey. This ensured that the EQ and SUS responses were collected under an identical scenario and could therefore be directly compared across experimental conditions. See Appendix B for specific statements and scales used in the EQ and SUS.

### 2.4. Procedure

Three immersive consumer studies assessing the engagement and usability of three different surveys were conducted. In each of the studies, participants evaluated three dip samples using one of the test surveys in the Department of Food Science and Technology’s Immersive Technologies Laboratory. This laboratory is equipped with a video wall and surround-sound system, used in conjunction to restore the environmental context during testing. A within-subject design was applied, and participants tested once per week for three weeks. To ensure that panelists did not lose interest in the evaluations or learn that the focus of the study was on the survey technology instead of the product, the type of dip varied across sessions. Dips were selected as the sample stimuli, as they were congruent with the chosen contextual environment for the study, a local Columbus cantina-style restaurant that was filmed on location. Presentation order of survey type, dip type, and samples within a dip type were randomized and counter-balanced across participants. This protected against possible confounding interactions between a dip type and survey type, as well as survey-order effects. 

Product questionnaires were created for each dip type (i.e., guacamole, salsa, and queso) for each survey technology. The number and types of test questions remained constant throughout the study, with changing product-specific attributes based on dip type. For example, consumers would be asked their opinion on the chunkiness of the salsas, whereas for the quesos, this attribute was changed to smoothness, as a chunky-cheese-based dip could indicate issues with product quality and lead to consumer distrust and feeling ill at ease, potentially negatively impacting participant engagement. Each product survey comprised typical questions a consumer would experience during a product evaluation, including liking/hedonic scales, Check All That Apply (CATA), attribute intensity rating scales, and Just About Right (JAR) scales. However, as the purpose of this research was to understand the effects of survey technology on participant engagement and usability, the specific product results are out of scope for this report. Instead, the technologies and modifications made to the various surveys will be discussed. 

The first survey, termed Control, was a traditional computer-based questionnaire presented on an iPad Air tablet with data collection through Compusense Cloud (Compusense Inc., Guelph, Ontario, Canada). Consumers were already familiar with this type of survey, as this is what is currently used in sensory and consumer evaluations in both OSU’s Food Perception and Liking Laboratory and the Sensory Evaluation Center. In this survey experience, participants read the questions and answer/scale options on the tablet screen and used their fingers to provide their responses. It was self-paced, and consumers could scroll down a page or move onto a subsequent section at their leisure. The only limitation was they could not move to a previous screen or sample, which is commonly encountered in typical computer-based sensory surveys to prevent changing responses. As this test questionnaire represents what is traditionally used, this survey was the first to be designed, and it was then used as the base of all test questionnaires for the study. 

The second survey, termed Translated, was presented using a third-generation Amazon Echo Dot with data collection through an industry collaborator’s proprietary platform (Aigora, Richmond, VA, USA). This questionnaire was considered a direct translation of the computer-based questionnaire into a verbal format. This meant that the test questions were asked in a similar manner to those in the Control survey, with only necessary modifications to allow for an auditory and verbal experience. In the Control survey, participants could read every scale point before selecting their responses. However, this was not possible when using a smart-speaker device, as reading aloud every scale point for every question would take too long and risk participants losing focus on the task. Instead, only the scale endpoints and corresponding numeric values were verbalized. For example, Alexa would ask a liking question by saying, “on a 9-point scale, with 1 being dislike extremely and 9 being like extremely, what is your overall opinion of this sample? (1–9 possible)”. A participant would then verbally provide their response with a number between the defined endpoints of the scale. Structuring questions this way allowed for maintaining the integrity of the Control survey while improving Alexa’s efficiency and hypothesized consumer experience. The difference in engagement and usability between the Translated and Control conditions provides insights into how only changing the device technology (i.e., tablet or smart speaker) affects the participant experience in immersive consumer testing. 

The last survey, termed Optimized, was also proctored using a third-generation Amazon Echo Dot, with data collection through Aigora’s platform. This questionnaire included hypothesized optimizations to the question format and response options thought to improve the participant test experience. The Optimized survey was tailored to have question structures that required simple word-based responses. This meant that most of the original scale questions were broken into several smaller questions that were answered with a binary response. For example, liking questions were broken into two separate questions. Participants were first asked, “did you like the sample?”, and based on the response (i.e., yes or no) were asked a follow-up question of “did you like it a lot?” if the initial answer was yes, or “did you dislike it a lot?” if the initial answer was no. For rating questions, Double-Faced Applicability [18] was used to assess the presence of an attribute and the participant’s confidence in their judgement. This approach followed the same multi-question structure but used both yes/no (for attribute presence) and sure/not sure (for confidence) responses. The basis for these proposed optimizations was from previous studies that found that participants better relate to or identify with word choices than numeric responses [19,20]. Moreover, responding verbally with words better simulates a conversational and more realistic experience than providing numeric answers. Comparing the engagement and usability results of the Translated and Optimized survey conditions allow us to understand whether current consumer testing surveys require further modification for smart-speaker delivery.

**Figure 1:**
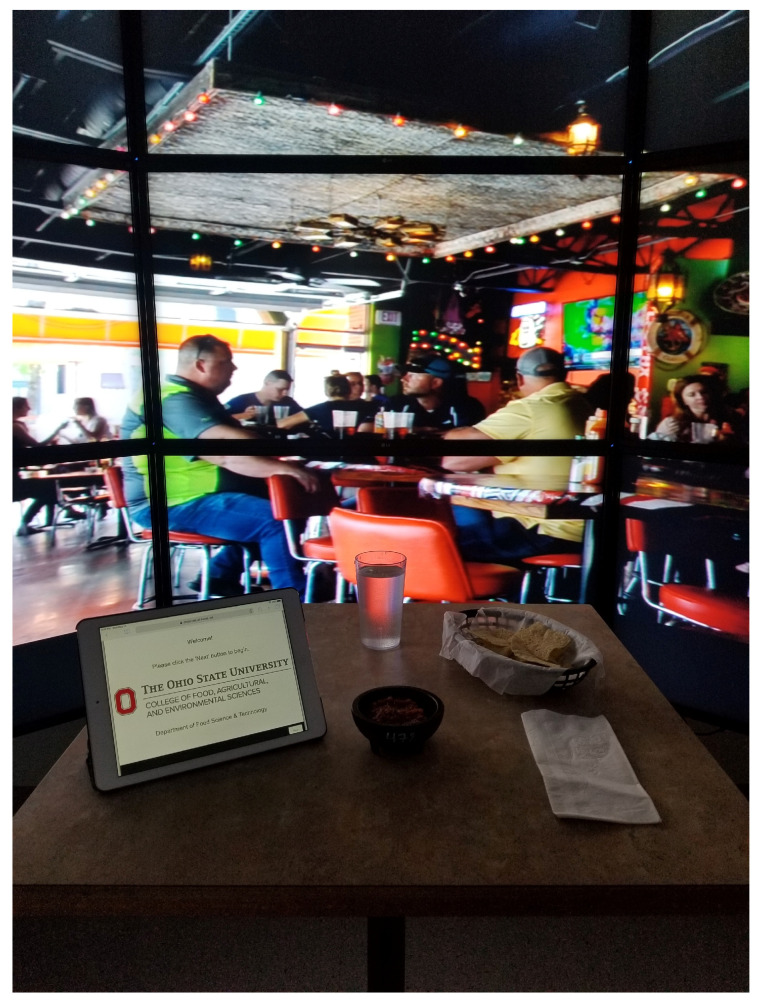
Example visual of testing space for computer-based-survey condition. For the smart-speaker-based surveys, the tablet was replaced with the appropriate Amazon Echo Dot.

During testing, participants were briefed on the study and then taken into the immersive cantina-style restaurant testing space with the video wall and audio already running. The test space consisted of a chip basket (Model NO. 1074, Tablecraft Product Co., Gurnee, IL, USA) lined with deli paper (Model SW-10, Durable Packaging International, Wheeling, IL, USA) and filled with unsalted tortilla chips (Meijer Inc., Grand Rapids, MI, USA). Unsalted tortilla chips were chosen to prevent possible cross-modal interactions between salt and a dip ingredient, as well as to serve as the palate cleanser for the study, as providing both unsalted crackers and tortilla chips would be unrealistic, possibly detracting from the context we aimed to create. Moreover, this ensured all participants used the same dip carrier, as some participants may have used the unsalted crackers in place of tortilla chips for dipping, creating an unequal comparison. The test space also had a glass of water (12 oz/355 mL, Tar-Hong Melamine USA, Inc., City of Industry, CA, USA), napkin, the survey device (either a smart speaker or tablet), and a card with the participant’s panel code. This code was unique to the participant and was used at each evaluation to allow for the proper compiling of the data for analysis. Figure 1 is a depiction of what the test space looked like for the Control survey condition. 

Once the panelist was seated, the test moderator prepared the first sample according to the rotation plan and brought it into the test area. This was performed by pouring/scooping the pre-measured 96 mL dip samples into the corresponding pre-labeled molcajete. For the queso samples, there was an additional 30 s heating step in the microwave (1100 W), as this category of dip is typically served warm. Upon receipt of the sample, the participant would start their survey if they were testing on a tablet. For the smart-speaker-based surveys, the test moderator started the product questionnaire for the participant by saying an invocation specific to the product category (for example, “Alexa, open guacamole survey”). The participant was instructed to perform a palate cleanse using water and unsalted tortilla chips before tasting the sample. They then went on to evaluate each sample by answering a series of questions focused on the product. After the participant finished evaluating the sample, the test moderator came to collect the leftover product while the participant performed a palate cleanse. In the tablet surveys, the palate cleanse was incorporated as a forced time break. In the smart-speaker surveys, each sample had a separate test questionnaire due to the current capabilities of the platform, so a palate-cleanse instruction was incorporated into the beginning of each individual questionnaire. The process was then repeated until all test samples were evaluated.

After the third sample was evaluated, the immersive testing system was turned off and a tablet was brought into the test space for the panelist to answer the EQ and SUS questionnaires. After the third test session, they were also asked to answer an exit questionnaire collecting usage, attitude, and demographic information. The exit questionnaire was asked after the EQ and SUS to prevent potential bias and memory effects.

### 2.5. Data Analysis 

Data analysis was performed using IBM SPSS Statistics version 27 (IBM Inc., Armonk, NY, USA). Before the data could be analyzed, both the EQ and SUS data needed to be appropriately treated. For the EQ, data were processed as outlined by Hannum et al. [13]. Individual agreement statements under each engagement factor (i.e., Affective Value, Purposeful Intent, and Active Involvement) were averaged so each panelist had three factor scores. The negative statements under Active Involvement were reverse-coded before averaging. A two-way Analysis of Variance (ANOVA) with Fisher’s Least Significant Difference (LSD) post hoc comparison was performed for each engagement factor and used to assess panel responses to the survey technology. Main effects were survey type (Control, Translated, and Optimized) and Judge (panelist), with the dependent variable being the engagement factor scores. 

For the SUS questionnaire, data were treated as outlined by Brooke [14]: positive statements were combined, negative statements were combined and reverse-coded, and the sum multiplied by a standard factor (2.5) to result in a single SUS score. A two-way ANOVA with Fisher’s LSD post hoc comparison was performed to assess usability of the different test surveys. Main effects were survey type (Control, Translated, and Optimized) and Judge (panelist), with the dependent variable being the SUS scores. 

Due to unplanned technical difficulties with the smart-speaker surveys during testing, additional analyses were conducted to understand whether participants were penalizing the smart-speaker technology when issues arose. Analyses were also performed to understand whether participants that experienced issues with the smart-speaker surveys were rewarding the tablet survey. However, due to the data being unequally distributed and a percentage of scores being tied between the two groups of consumers (i.e., those that experienced issues and those that did not), a non-parametric test was employed. A Wilcoxon–Mann–Whitney Rank Sums Test was used to compare the engagement and usability scores of participants that experienced technical issues (i.e., were dropped/had their survey end prematurely) and those that did not for both penalizing and rewarding the various survey technologies. The z-scores derived from this type of analysis were used to determine whether scoring patterns between the two participant groups were significantly different for both engagement and usability.

All mean scores are presented as the mean ± the standard error. 

## 3. Results

### 3.1. Assessment of Survey Technology Effects on Engagement and Usability 

As we were interested in assessing the effect of the questionnaire type on engagement and usability, panelists that experienced technical difficulties (i.e., they were dropped from the survey due to the Echo Dots disconnecting from the network) during one of their smart-speaker surveys had their scores imputed with the panel mean for the survey they were dropped from. This ensured that experiences with technical anomalies did not falsely inflate or deflate the engagement and usability metrics. A total of 11 of the 57 panelists had their data imputed for the Translated survey, and 12 of the 57 panelists had their data imputed for the Optimized survey. Participants that experienced technical difficulties in both of their smart-speaker-based surveys (N = 4) did not have their data imputed for two reasons: one, they did not experience a flawless smart-speaker scenario to which they could compare, and two, because the technology itself was being assessed and performed with the same level of reliability in both experimental conditions, there was no need to treat their data differently than those of participants who also experienced the same level of quality in both smart-speaker treatments and did not drop from either survey. For this reason, this section is divided into findings using imputed data and results comparing the engagement and usability metrics of participants that experienced a drop from the survey and those that did not.

#### 3.1.1. Survey Technology Effects on Engagement

**Figure 2:**
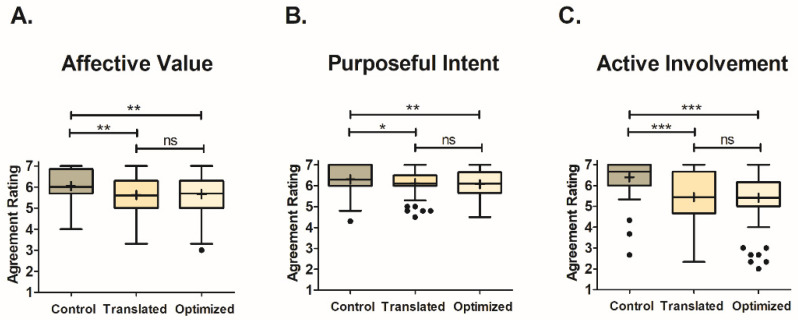
Box-and-Whisker plots of engagement mean factor ratings for each survey type (Control, Translated, and Optimized) grouped by engagement factor ((**A**) for Affective Value, (**B**) for Purposeful Intent, and (**C**) for Active Involvement). Mean scores are represented by “+” signs and median scores by horizontal lines within the boxes. The interquartile range is represented by the height of the boxes. Tukey’s method was used to calculate the whiskers and outliers (represented as solid black dots). Outliers were identified for observational purposes and therefore included in all analyses. Significant differences between the survey types were determined through Fisher’s LSD post hoc comparison and are shown as * for *p* < 0.05, ** for *p* < 0.01, and *** for *p* < 0.001. Results that were not significant are shown as “ns”.

The Control condition received significantly higher (Affective Value: F_2,56_ = 7.31, *p* = 0.001; Purposeful Intent: F_2,56_ = 4.88, *p* = 0.009; Active Involvement: F_2,56_ = 25.69, *p* < 0.001) engagement scores (Affective Value: X = 6.1 ± 0.1; Purposeful Intent: X = 6.3 ± 0.1; Active Involvement: X = 6.4 ± 0.1) across all engagement factors compared to both the Translated (Affective Value: X = 5.6 ± 0.1; Purposeful Intent: X = 6.1 ± 0.1; Active Involvement: X = 5.4 ± 0.2) and Optimized (Affective Value: X = 5.7 ± 0.1; Purposeful Intent: X = 6.1 ± 0.1; Active Involvement: X = 5.4 ± 0.2) smart-speaker surveys. Figure 2 portrays the Box-and-Whisker plots for the engagement scores of each condition, grouped by engagement factor, with whiskers and outliers calculated using Tukey’s method. Although significant differences were found between the Control survey and the smart-speaker surveys, the smart-speaker surveys did not significantly differ from one another for any of the engagement factors. Looking within a survey type, the Control condition had the greatest mean engagement score for Active Involvement. Conversely, both smart-speaker surveys received their lowest engagement scores for Active Involvement, with their highest mean engagement scores being for Purposeful Intent.

Regardless of the survey type, the interquartile range was the smallest for Purposeful Intent (Figure 2B). However, the Translated condition had its greatest number of outliers for this factor (six participants represented as solid black dots). The Control and Optimized conditions both had their greatest number of outliers for Active Involvement (three for Control and seven for Optimized). It should be noted that, for this study, the use of the term outlier does not indicate a poor or misaligned performance, as there were no correct/incorrect responses for the metrics in question, as they were based on the participants’ opinions of their experiences. Instead, outliers are solely for observational purposes to indicate participants that felt differently about their experience for a given survey type. Therefore, outlier data were included in all analyses. 

#### 3.1.2. Survey Technology Effects on Usability

**Figure 3:**
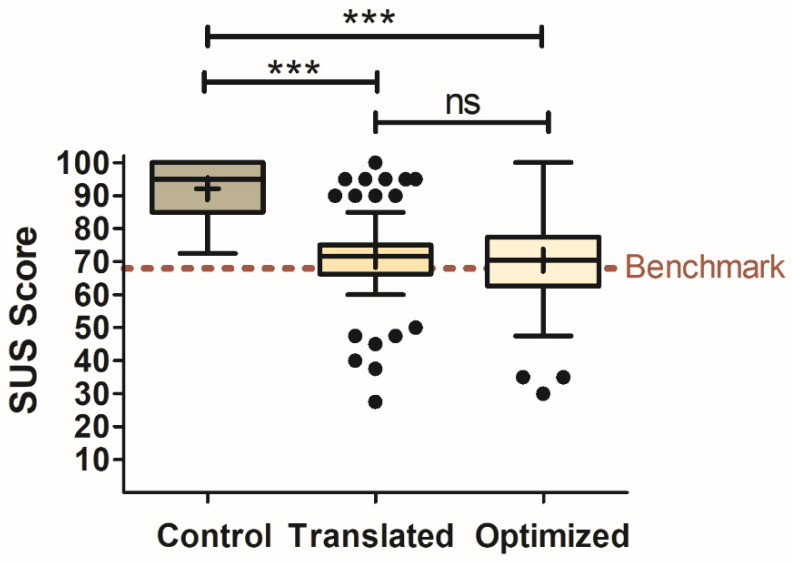
Box-and-Whisker plots of SUS ratings for each survey type (Control, Translated, and Optimized). SUS Benchmark score for average usability is shown at y = 68 as a dashed red line. Mean scores are represented by “+” signs and median scores by horizontal lines within the boxes. The interquartile range is represented by the height of the boxes. Tukey’s method was used to calculate the whiskers and outliers (represented as solid black dots). Outliers were identified for observational purposes and therefore included in all analyses. Significant differences between the survey types were determined through LSD post hoc comparison and are shown as *** for *p* < 0.001. Results that were not significant are shown as “ns”. The benchmark value of 68 out of 100 is the 50th percentile score obtained from a meta-analysis of 400+ systems previously evaluated to indicate an average usable system [15,16].

The Control condition scored significantly higher (F_2,56_ = 66.31, *p* < 0.001) in usability than both smart-speaker-based survey conditions. Figure 3 displays the results of the SUS scores for each of the survey types. The mean score for the tablet (92.1 ± 1.1) was well above the benchmark for a usable technology (i.e., SUS score of 68). Even though the smart-speaker surveys had significantly lower SUS scores compared to the Control, they were both above the benchmark for being a usable technology. The Translated survey had a mean score of 71.6 ± 2.0, while the Optimized survey had a mean score of 70.4 ± 2.1. There was no significant difference found between the two smart-speaker survey types. Similar to the engagement findings, there were also outlying participants for both smart-speaker surveys. The Translated survey had outlying participants both above (10) and below (7) the interquartile range. The Optimized condition only had outliers below the interquartile range (3). 

### 3.2. Assessment of Panelist Response to Smart-Speaker-Survey Technical Difficulties 

To understand whether panelists that experienced a drop (i.e., survey disconnected before finishing a sample evaluation) during testing penalized the smart-speaker-survey technology, separate analyses comparing dropped participants with those that did not experience technical issues during testing were performed. Panelists that were dropped from only one survey were categorized as “drop” (N = 11 for Translated and N = 12 for Optimized), while participants that either did not experience a drop (N = 30) in either survey or were dropped from both surveys (N = 4) were classified as “no drop” based on the previously presented rationale. 

#### 3.2.1. Effect of Smart-Speaker-Survey Technical Difficulties on Engagement

**Figure 4:**
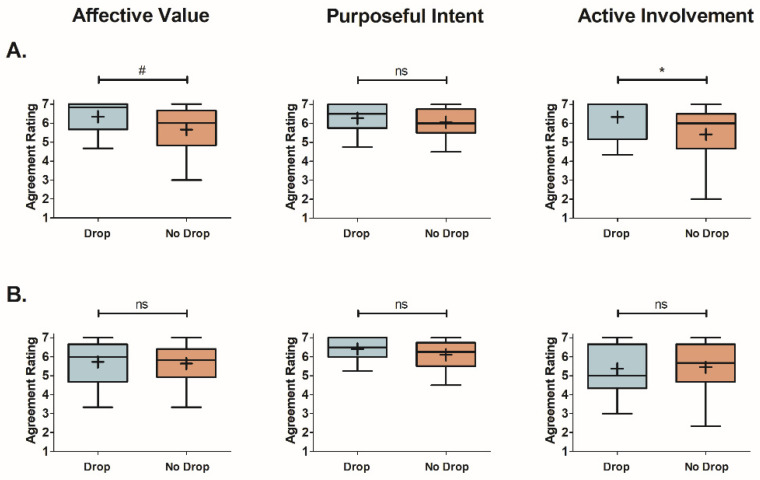
Box-and-Whisker plots comparing engagement mean factor ratings for participants that experienced a drop during testing and those that did not. Findings are displayed for each smart-speaker survey ((**A**) for Optimized, (**B**) for Translated) and grouped by engagement factor (left pane for Affective Value, middle pane for Purposeful Intent, and right pane for Active Involvement). Mean scores are represented by “+” signs and median scores by horizontal lines within the boxes. The interquartile range is represented by the height of the boxes. Significant differences between the survey types were determined through Wilcoxon–Mann–Whitney Rank Sums Test and are shown as * for *p* < 0.05. Marginally significant results represented as # for *p* = 0.056. Results that were not significant are shown as “ns”.

The results of the comparison between participants that experienced a drop during a smart-speaker evaluation and those that did not are displayed in Figure 4. Of the two smart-speaker surveys, the Optimized condition had a significant difference in Active Involvement (z = 2.24, *p* = 0.025, two-tailed) between drop (X = 6.3 ± 0.3) and no-drop (X = 5.4 ± 0.2) participants (Figure 4A). A marginally significant difference was found for Affective Value between these participant groups (z = 1.91, *p* = 0.056, two-tailed; drop: X = 6.3 ± 0.3; no drop: X = 5.7 ± 0.2), while no difference was observed for Purposeful Intent (z = 0.85, *p* = 0.395, two-tailed; drop: X = 6.3 ± 0.2; no drop: X = 6.1 ± 0.1). Participants that experienced a drop gave significantly higher ratings for Active Involvement and marginally higher ratings for Affective Value compared to those that did not experience a drop. These findings indicate that, despite experiencing technical difficulties during the Optimized survey, participants who were dropped were surprisingly more engaged than those that did not run into any technical issues.

Unlike the Optimized survey, the Translated condition did not see a difference in ratings between participants that experienced a drop (Affective Value: X = 5.7 ± 0.4; Purposeful Intent: X = 6.4 ± 0.2; Active Involvement: X = 5.4 ± 0.4) and those that did not (Affective Value: X = 5.6 ± 0.2; Purposeful Intent: X = 6.1 ± 0.1; Active Involvement: X = 5.4 ± 0.2) for any engagement factor (Affective Value: z = 0.36, *p* = 0.719, two-tailed; Purposeful Intent: z = 1.17, *p* = 0.242, two-tailed; Active Involvement: z = 0.16, *p* = 0.873, two-tailed).

#### 3.2.2. Effect of Smart-Speaker-Survey Technical Difficulties on Usability

**Figure 5:**
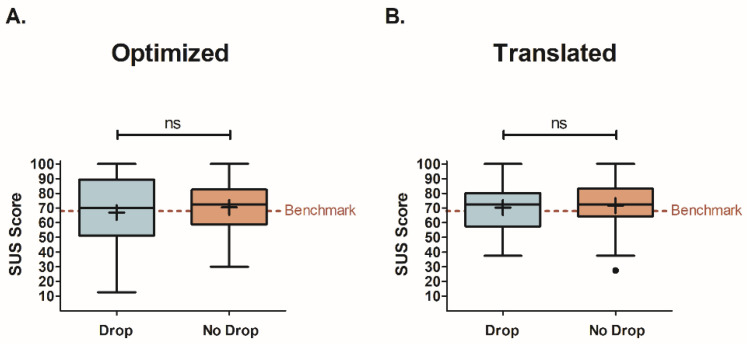
Box-and-Whisker plots comparing SUS ratings for participants that experienced a drop during testing and those that did not. Findings are displayed for each smart-speaker survey ((**A**) for Optimized, (**B**) for Translated). SUS Benchmark score for average usability is shown at y = 68 as a dashed red line. Mean scores are represented by “+” signs and median scores by horizontal lines within the boxes. The interquartile range is represented by the height of the boxes. Tukey’s method was used to calculate the whiskers and outliers (represented as solid black dots). Outliers were identified for observational purposes and therefore included in all analyses. Significant differences between the survey types were tested via Wilcoxon–Mann–Whitney Rank Sums Test. Results that were not significant are shown as “ns”. The benchmark value of 68 out of 100 is the 50th percentile score obtained from a meta-analysis of 400+ systems previously evaluated to indicate an average usable system [15,16].

No significant differences were found in the SUS scores between drop and no-drop participants for either the Translated (z = 0.10, *p* = 0.920, two-tailed; drop: X = 70.2 ± 6.1; no drop: X = 71.6 ± 2.5) or Optimized (z = 0.19, *p* = 0.849, two-tailed; drop: X = 66.9 ± 7.6; no drop: X = 70.4 ± 2.6) smart-speaker surveys, as shown in Figure 5.

### 3.3. Assessment of Panelist Rewarding Control Surveys

Another question that arose from the study was whether participants who experienced a drop rewarded the tablet-based survey, meaning they inflated their engagement and usability scores for the Control condition. A separate group of analyses was conducted to address this question. Participants that experienced a drop before their tablet condition were categorized as “drop” (N = 13), while participants that either did not experience a drop (N = 30) or dropped in both smart-speaker-based surveys after their Control condition (N = 3) were categorized as “no drop”. Panelists that experienced a drop in only one smart-speaker-based survey after they completed their Control survey were not included in this analysis. This was for two reasons: one, these participants could not use their negative experience to reward a past survey, and two, as they experienced only one drop that may have influenced their engagement and usability ratings, they could not be considered “no drop”. As such, 46 of the 57 participants were included in these analyses.

#### 3.3.1. Rewarding Control Condition for Engagement

**Figure 6:**
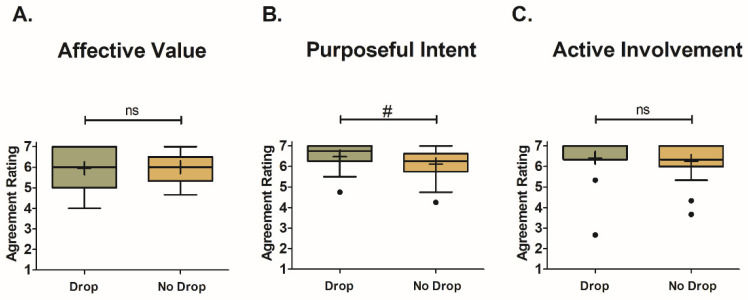
Box-and-Whisker plots comparing engagement mean factor ratings for participants that experienced a drop during smart-speaker testing and before the Control survey and those that did not (N = 46). Findings are displayed for the Control survey and grouped by engagement factor ((**A**) for Affective Value, (**B**) for Purposeful Intent, and (**C**) for Active Involvement). Mean scores are represented by “+” signs and median scores by horizontal lines within the boxes. The interquartile range is represented by the height of the boxes. Tukey’s method was used to calculate the whiskers and outliers (represented as solid black dots). Outliers were identified for observational purposes and therefore included in all analyses. Significant differences between the survey types were tested via Wilcoxon–Mann–Whitney Rank Sums Test. Marginally significant results represented as # for *p* = 0.056. Results that were not significant are shown as “ns”.

Of the three engagement factors, only Purposeful Intent had a marginally significant difference (z = 1.91, *p* = 0.056, two-tailed) between participants that experienced a drop (X = 6.5 ± 0.2) and those that did not (X = 6.1 ± 0.1). This meant that panelists who experienced a drop in one of their smart-speaker surveys before their Control condition gave the tablet slightly higher scores for this engagement factor. No difference was observed between the scoring patterns of the two groups of participants for Affective Value (z = −0.04, *p* = 0.968, two-tailed; drop: X = 6.0 ± 0.3; no drop: X = 6.0 ± 0.1) or Active Involvement (z = 1.41, *p* = 0.159, two-tailed; drop: X = 6.4 ± 0.3; no drop: X = 6.3 ± 0.1). Figure 6 depicts the comparison between panelists that experienced a drop, those that did not, and the effect this had on the Control-condition scores.

#### 3.3.2. Rewarding Control Condition for Usability

**Figure 7:**
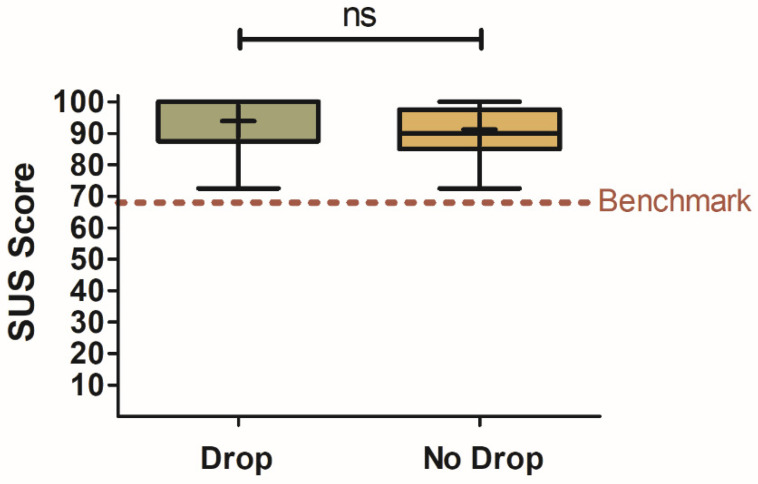
Box-and-Whisker plots comparing SUS ratings for participants that experienced a drop during smart-speaker testing and before the Control survey and those that did not (N = 46). Findings are displayed for the Control survey. SUS Benchmark score for average usability is shown at y = 68 as a dashed red line. Mean scores are represented by “+” signs and median scores by horizontal lines within the boxes. The interquartile range is represented by the height of the boxes. Significant differences between the survey types were tested via Wilcoxon–Mann–Whitney Rank Sums Test. Results that were not significant are shown as “ns”. The benchmark value of 68 out of 100 is the 50th percentile score obtained from a meta-analysis of 400+ systems previously evaluated to indicate an average usable system [15,16].

Unlike engagement, there was no significant difference found between the two consumer groups and their subsequent usability ratings for the Control condition (z = 1.44, *p* = 0.150, two-tailed), as shown in Figure 7. Both participants that experienced a drop in a smart-speaker questionnaire (X = 93.8 ± 2.5) and those that did not (X = 91.1 ± 1.4) scored the tablet as being highly usable.

## 4. Discussion

### 4.1. Assessment of Survey Technology Effect on Engagement and Usability

#### 4.1.1. Survey Technology Effects on Engagement

Findings from this study disproved our hypotheses regarding the panelist engagement increasing with the use of smart-speaker-survey technology in immersive consumer testing. Instead, the opposite result was observed, with the Control tablet condition being rated as significantly more engaging for all engagement factors. There were also no differences found between the Translated and Optimized smart-speaker surveys, indicating that the proposed questionnaire optimizations were not robust enough to improve the user experience. The initial hypotheses for the research were derived from the idea that smart speakers would create a more interactive and conversational testing environment, leading to a more realistic experience and increased engagement. However, strict rules were required for how to interact with Alexa, which only increased the complexity of the evaluation. 

In the smart-speaker surveys, participants were restricted in how they could respond to questions and the length of time they could spend on a given question. For certain questions, participants had to say specific statements before their response to signal Alexa to record the answer. For example, participants had to begin their open-comment statement with the phrase “I thought” or an error message would play. Moreover, Alexa would provide answer options for questions, and if a slight variation in the answer option was verbalized, then Alexa would respond with an error message and not record the response. Time constraints were imposed for the open-comment questions and the amount of time panelists had to provide an answer. For open comments, Alexa would stop recording and interrupt the panelist if a brief pause (such as taking a breath in between statements) was detected. If a panelist was able to verbalize several opinions about the sample without taking a breath, then Alexa would often not record the response and just repeat the question. Participants that experienced issues with their open comments either shortened their comments on subsequent samples or skipped the question altogether. Open comments provide participants with an opportunity to give additional insights on product aspects that are not captured in the closed-ended questions of a survey. If participants opt to skip such questions, then the findings from the consumer test could be compromised and indicate a product is ready to launch when in reality it is not. When responding to closed-ended questions, participants had from 10 to 15 s to provide an answer. If they did not provide an answer within the time limit, then Alexa would repeat the question and then disconnect from the survey if the participant again took time to think over their answer options. The strict rules for interacting with Alexa added undue stress to the evaluation and forced participants to keep track of these nuances during the sample evaluation. This could have detracted from the overall evaluation and led to a less focused and engaging experience.

The absence of complex guardrails in the Control survey may be one reason this condition was significantly more engaging than both smart-speaker surveys. The only imposed requirements for the tablet questionnaire were that panelists needed to enter their panel code to begin the evaluation, they had to wait 30 s in between samples before being allowed to move on to the next sample, and they could not revisit previously answered questions. Other than this, participants had complete control over their survey experience. Having personal control over the survey allowed panelists to take a more active/proactive role in their evaluation, leading to an increased sense of responsibility, purpose, and overall engagement [21]. The smart-speaker surveys forced the panelists into a passive or reactive role, as they were at the whim of Alexa’s pacing and intricacies, resulting in participants zoning out and assigning lower Active Involvement scores. Participants taking a more proactive role in their evaluation could also explain why the Control survey had the highest mean score for Active Involvement. Panelists had the opportunity to shift their focus between the samples, the questionnaire, and the immersive environment at their leisure to prevent becoming bored with the task. They were also in charge of setting the pace of their survey experience and could move through the evaluation at a faster or slower speed to ensure they remained focused on the evaluation.

Anecdotal evidence from open comments on the survey experience provided additional insights into why the smart-speaker surveys were less engaging than the Control survey. Several panelists mentioned they had a fear of verbalizing their thoughts and being judged for their opinions knowing a test proctor was sitting behind the one-way mirror in the control room. Fear of judgement could cause anxiety and make it difficult to focus, leading to disengagement [22,23]. The Control survey allowed panelists to conceal their responses and provide more honest opinions/ratings [24,25], easing participants that stress easily. Some participants also felt that the time pressure associated with the smart-speaker surveys detracted from the evaluation and made it difficult to focus, a trend seen in other studies involving time constraints [17,26]. Another group of panelists felt that the smart-speaker surveys were difficult to use in the immersive space due to environmental distractions. The audio and visuals from the video wall made it difficult to focus on what Alexa was saying, indicating that the use of smart-speaker surveys in an environment with too many external cues could lead to cognitive overload and disengagement [27,28]. Consumers did not indicate any issues with sensory overload when taking the Control survey, suggesting that this may be another reason they felt more engaged.

Panelists may also have been more engaged in the Control condition due to familiarity and established trust with the technology. Participants may have known from prior experience that the tablet was reliable and would not disconnect from the test. When they experienced an issue with the smart-speaker surveys, panelists felt that they were responsible and felt remorse. This guilt could have negatively impacted the participant engagement in the smart-speaker questionnaires, leading to higher engagement scores for the Control questionnaire. Additionally, the Control survey allowed for multiple samples to be evaluated using the same questionnaire. Current smart-speaker-survey capabilities do not allow for multi-sample testing, requiring a separate survey for each sample. This confused some panelists as to why they were ending a survey after evaluating only one product. This sense of confusion and uncertainty was absent from the Control condition, potentially leading to higher engagement scores.

Lastly, the smart speakers may have been less engaging than the Control survey due to a phenomenon known as the “uncanny valley”. The uncanny valley relates to the relationship between a technology’s resemblance to human characteristics and the emotional reaction to said technology [29]. In general, as a technology becomes more realistic, the liking of it increases. This is true up to a certain point. While there is no constant definition for what creates the change in opinion, there is a point where the liking of a technology drastically decreases when it starts to approach being too realistic yet contains minor flaws that serve as a reminder it is not human. The disconnect between realistic human character and knowing the technology is not human creates a feeling of mistrust. In the case of the smart-speaker surveys, it is possible that the combination of Alexa and the immersive test setting approached a realistic experience to the point that it fell into the uncanny valley. In doing so, participants may have felt a sense of uneasiness resulting in decreased engagement.

While the smart-speaker surveys were equally less engaging compared to the Control survey, the question of why they received similar engagement scores even though they used different question structures and scales remained. This is likely due to the two survey experiences having more in common with one another than they did differences. In both cases, the surveys involved auditory and verbal components that had to compete with the environmental context of the immersive testing space. During testing, there were still too many rules for how to interact with Alexa, placing impositions on the participants. Responses in both conditions, whether numeric or word-based, had to be verbalized in a rigid format for responses to be recorded. At this time, the proposed survey optimizations were not great enough to create a difference between the two smart-speaker surveys, as the programming behind the smart-speaker technology was not advanced enough to create a truly conversational experience. As both smart-speaker surveys were subject to the same test nuances, it makes sense that they would have similar trends in the resulting engagement factors. 

All surveys had outlying participants that gave lower engagement scores than the rest of the panel. The Translated survey had the greatest number of outliers give lower engagement scores for Purposeful Intent. This result may have been due to requiring a verbal numeric response. Some participants have difficulty relating to arbitrary numbers [20], while others have difficulty recalling the scales without having them visually available. Specifically for this survey, some participants confused the numeric scales for different question types (i.e., rating scales used 0–3, while JAR scales used 1–5), leading to incorrect responses and Alexa error messages with reminders of the scale. This could have led to an annoyance or frustration with the task, resulting in discouragement and lower Purposeful Intent scores. 

The remaining Control and Optimized surveys also had outlying participants with lower engagement scores; however, these were for Active Involvement. For the Control survey, some participants completed the evaluation in one-third of the time it took to complete a smart-speaker evaluation (i.e., approximately 10 min). These participants may have been less focused on the task itself and more on completing the task as quickly as possible to make a prior appointment, such as returning to work, or to leave before it started raining on stormy days. It is also possible that these panelists became bored with answering the same questions for all test samples or disinterested due to disliking the smokey modification made to the samples. For the Optimized condition, the increased questionnaire length due to branched survey logic for two-part questions and repetitive answer options could have caused some panelists to zone out or experience mental fatigue. This could have resulted in lower Active Involvement engagement scores. 

#### 4.1.2. Survey Technology Effects on Usability

Our hypothesis for the Control survey receiving higher SUS ratings than both smart-speaker surveys was supported by the data. The Control survey scored above the average benchmark of 68 for usability [15,16]. This may be due to participants not experiencing any issues with the tablet during testing. Compusense data collection software has an established user interface optimized for the consumer experience. Participants also had prior experience with the Compusense user interface, as all participants had previously participated in sensory and consumer testing.

Both smart-speaker surveys received significantly lower SUS scores compared to the Control condition, rated just above the benchmark for being a usable technology. This was likely due to the rigid rules of interacting with Alexa. Specific phrases were required to have an answer option recorded and move through the survey. If the information was not verbalized correctly, then Alexa would respond with an error message and the appropriate answer options. There were timing intricacies with open comments and question responses that had to be followed or the survey would disconnect. When the survey dropped, the researcher needed to restart the survey and skip through all the questions (and possible questions from the survey logic), as there was no way to skip to a specific question. Participant codes were four-digit numbers and could be said as a whole number or four separate digits, but sample codes, which were three-digit numbers, had to be said as whole numbers. This caused confusion, as panelists had to remember when and how to say which code. Participants would sometimes also give incorrect responses that would invoke error messages or sometimes prematurely end the survey. During the Translated survey, participants sometimes confused the intensity rating scales with the JAR scales and would not discover this until they used an incorrect number and received an error message. For the Optimized survey, participants had issues keeping track of the JAR-scale options (i.e., not enough, just about right, and too much), with some participants needing to write down the options to remember what to say. For this question, participants would often remember the term “enough” and would try using this in place of “just about right”. Unfortunately, “enough” was a stop code for Alexa and would immediately end the survey, requiring the researcher to restart the test and skip through to where it left off. 

While both smart-speaker surveys were significantly different from the Control survey, they were not different from one another. This was because both smart-speaker surveys had the same nuances (mentioned above) that complicated testing. The smart-speaker surveys were not conversational enough. Instead, Alexa was monotonous and did not allow for free responses, which could have stifled its usability. When a panelist said what they wanted, they received an error message until they conformed to the system’s specific codes. Moreover, the hypothesized improvements to the Optimized survey were not robust enough to warrant a difference. Word-based responses were selected, as people better relate to words than numbers [30]. However, the selected words were purposefully kept very simple, which, in turn, could have decreased their relatability and the effect of the proposed optimization.

As with the engagement results, there were several outlying participants that differed in their SUS ratings for the Translated and Optimized surveys. The Translated surveys had outliers both above and below the whiskers in the Box-and-Whisker plot. This suggests that participants had less agreement on this survey compared to the others. Some may have liked providing verbal responses, using numeric scales, and having fewer questions than the word-based survey. Others felt the opposite and therefore gave lower SUS ratings. For the Optimized survey, the only outliers were those that gave much lower SUS scores. These were most likely participants that provided incorrect JAR responses or felt that the survey had too many questions.

### 4.2. Assessment of Panelist Response to Smart-Speaker-Survey Technical Difficulties

#### 4.2.1. Effect of Smart-Speaker-Survey Technical Difficulties on Engagement 

Panelists that were dropped from the Optimized survey were significantly more engaged than those that were not, specifically in Active Involvement. This was likely due to the participants having increased interaction with a person (i.e., the researcher) compared to those that did not drop. During the test briefing, participants were reminded that inactivity or waiting too long to respond would lead to the smart speaker disconnecting. When a drop occurred, the test proctor needed to enter the test space and restart the survey. The researcher was very apologetic for the inconvenience and stayed with the participants until they knew the survey would not drop again. In turn, most participants were remorseful and expressed guilt, as they felt responsible for causing an issue. It is possible that these participants could have overcompensated in subsequent sample surveys by being more attentive to the task. Other participants mentioned they were used to Alexa being unreliable at home, which mitigated feelings of frustration and disengagement. 

Participants that experienced a drop from the Optimized survey were also marginally more engaged in Affective Value. This engagement factor relates to a panelist’s interest in and enjoyment experienced during a task. Again, this could be explained by the increased human interaction with the researcher, or by simply being given extra time to sit in an immersive environment and consume a product category they enjoy. As these results were marginally significant, these trends should be noted but taken with caution. 

Active Involvement was the only engagement factor with a clear significant difference, indicating that this factor may be susceptible to sudden test scenario changes, while Affective Value and Purposeful Intent were more stable. Active Involvement relates to a participant’s focus and activity in a task. When participants zoned out in the smart-speaker scenarios, a survey drop served as a break from the monotony of the Alexa survey and recaptured the participant’s focus on the task at hand. Affective Value and Purposeful Intent relate to the overall enjoyment in and purpose for the completion of the task, respectively, which serve as overarching themes during testing and remain constant, regardless of sudden and brief changes in the test scenario. For example, in the immersive environment, Alexa’s characteristics, and the degree of interaction with Alexa, remained constant throughout the test, as did the motivation for helping a graduate student or receiving monetary compensation for completing the evaluation. 

Participants that dropped from the Translated survey did not change their engagement scores, indicating that the changes made to the Optimized survey may have influenced engagement differently than expected. The Optimized survey was the longest of the three due to the necessary question branching increasing the number of responses a participant needed to provide. Those that did not drop from this survey may have become bored with the task, resulting in disengagement. The Translated survey did not have this issue, as it did not require questions to be asked in multiple parts; thus, participants that did not experience a drop did not have as much time to lose interest in the task. Moreover, when a drop occurred in the Translated survey, it took less time to skip through the questions to where the survey left off, making a drop seem less problematic than in the Optimized condition. The trade-off in creating a smart-speaker survey that allowed for word-based responses was an increase in the panelist time commitment; yet, the results of this analysis indicate that a break during the survey could positively impact participant engagement. However, it is uncertain how this would influence other metrics, such as the panelist performance.

#### 4.2.2. Effect of Smart-Speaker-Survey Technical Difficulties on Usability

Unlike engagement, there were no significant differences in the SUS ratings between participants that experienced a drop from a smart-speaker survey and those that did not. This could be due to the panelists in the study being familiar with Amazon Alexa devices dropping in the middle of performing a task. During the study, Amazon released a new program called Amazon Sidewalk with the aim of stabilizing Alexa-powered devices and their network connections [31]. This indicates that Alexa experiencing connectivity issues and disconnecting is a known issue. Another reason for the lack of significant differences between participants that experienced a drop and those that did not is that rating the usability of a technology is an objective assessment and less prone to emotional or subjective influence, unlike engagement [32]. 

### 4.3. Assessment of Panelist Rewarding Control Surveys

#### 4.3.1. Rewarding Control Condition for Engagement

Participants that experienced a drop in a smart-speaker survey prior to taking the Control survey scored the tablet marginally higher in Purposeful Intent than those that did not experience a drop. The dropped participants may have come to appreciate the ease and reliability of the familiar tablet experience and therefore assigned slightly higher ratings due to reclaiming control over the evaluation. The Control condition had participants take a more proactive role in their evaluation and established a sense of responsibility. The tablet required participants to use their own senses to facilitate the survey at their own pace. When participants have an increased sense of ownership in a task, they may have more dedication to complete the task, and therefore more purpose to complete the evaluation [21]. Smart-speaker-dropped participants did not reward the Control condition for Affective Value or Active Involvement, suggesting that these engagement factors may better relate to experiences within a survey condition and are less influenced by prior survey experiences.

#### 4.3.2. Rewarding Control Condition for Usability

Participants that experienced a smart-speaker-survey drop prior to participating in the Control condition did not reward the tablet for usability. The tablet functioned as expected and provided a consistent test experience within the survey type, regardless of how the smart-speaker surveys behaved. This suggests that SUS ratings are not influenced by previous experiences with different survey technology and the objective assessment of the tablet performance was independent of the other survey technologies explored in this study. The difference in the reward of the Control condition between usability and engagement provides further rationale for the previously mentioned argument that rating a technology is objective and less likely to be influenced by experiences with other technologies. This is the opposite of engagement, which is susceptible to subjectivity.

### 4.4. Future Directions

The findings from this study provide insights into the current capabilities of smart-speaker surveys for in-hall immersive consumer testing. As this was the first study of its kind, it is expected that further smart-speaker-survey improvements will be necessary to increase panelist engagement and device usability. However, the smart-speaker technological issues experienced in this study indicate that more research needs to be performed on the Amazon Web Services (AWS) operating system. The root cause for the smart-speaker surveys disconnecting from the network remains an enigma. Before conducting smart-speaker research, it is recommended to work with an AWS coordinator at a place of business to determine the potential for smart-speaker devices dropping from the network and implement solutions to prevent this. At the very least, an interim solution is developing the capability to skip to a specific question so that participants do not have to sit and verbally skip through every prior question before resuming the test.

Further research and optimizations in Alexa’s programming should be explored to create a truly conversational evaluation experience. The strict rules imposed on the participants for how to respond and record answers detracted from the immersive experience. Added complexity and requiring panelists to keep track of Alexa’s additional nuances served as a stressor and distraction. If participants forgot certain rules, the investigator was in the next room and could run in to assist; however, this would not be possible in an in-home test scenario. Smart-speaker devices naturally fit with in-home testing to capture in-the-moment product experiences, but if consumers run into issues while testing at home, they would not have anyone available to troubleshoot the survey. This could lead to incomplete evaluations and poor data quality. While smart-speaker technology will undoubtedly continue to progress in survey execution capabilities, sensory scientists need to be mindful in selecting questions with minimal rules that all consumers could easily answer. For example, current capabilities may not provide a useful open-comment experience for consumers, perhaps creating the need to use a tablet in conjunction with a smart speaker to capture these spontaneous insights.

Future studies should also further investigate participant behavior beyond engagement and usability when using smart-speaker surveys. Participants commented that using a smart-speaker survey in the immersive laboratory was distracting and led to sensory overload. While smart-speaker surveys and their current capabilities may not be suited for an immersive testing environment with competing audio, it is possible that these surveys may improve panelist engagement in traditional in-hall testing scenarios (i.e., sensory testing booths). It would also be of interest to use eye-tracking technology to understand where participants are looking when providing verbal responses. Some participants looked at Alexa when providing an answer option instead of paying attention to the video wall. This may indicate that participants feel more comfortable directing their answers to who is asking the question instead of speaking to an empty space, which can assist in determining the placement of smart speakers in a testing environment. Eye-tracking technology can also be employed to explore the influence of visual stimulation from the environmental context on sensory overload during smart-speaker testing. It would also be interesting to measure the amount of product consumed during the evaluation. Some participants ate more of their sample, even when they disliked it, when using a hands-free smart-speaker survey compared to the tablet condition. Product experiences and opinions can change depending on the amount of product consumed. To gain product insights that better reflect consumption in everyday life, smart-speaker surveys could assist with participants consuming more of the sample (either through re-tasting instructions or simply because it is a hands-free experience). Moreover, as technological advances improve, smart-speaker surveys can be combined with other systems (such as virtual reality or extended reality) to provide a solution for data collection in fully immersive scenarios that would be more representative of real-life situations. 

Lastly, it would be of interest to understand the investigator’s experience in using smart-speaker surveys. As sensory scientists and technicians would be the ones to execute smart-speaker in-hall studies, they could provide insights into what works well and what needs to be improved. If there are many issues, then the benefits of smart-speaker surveys would be overshadowed, and researchers may opt to use tried-and-true computer-based questionnaires. In the present study, every time a smart-speaker survey was about to be tested, researchers felt a sense of dread in knowing that there were most likely going to be issues with the test experience. Improvements to Alexa’s conversational style and addressing the root cause of smart-speaker-survey drops would reduce researcher test anxiety; however, until then, it is important to capture the researcher’s point of view to quickly address anything that can currently be optimized.

### 4.5. Limitations of the Research

While this study provided key learnings and considerations for immersive consumer testing with smart speakers, there are some limitations to the current research. Amazon Alexa users were chosen for the study because they were already familiar with the platform and nuanced temperament of the smart-speaker devices. However, it is unknown how these new survey styles and their technological issues would be received by non-Alexa smart-speaker users and those that do not use smart speakers. It is possible that the engagement and system usability findings would not apply to these groups. Moreover, the chosen environment of a cantina-style restaurant was a social atmosphere and could be loud/overstimulating for some participants. Selecting an environmental context that lacks a social aspect (i.e., a quieter and less busy setting, such as in the home or at a park) could change the interaction with Alexa and overall experience. Lastly, a traditional booth or non-immersive setting was not included in this study, so it is unknown whether the engagement scores for the smart-speaker surveys were inflated due to the immersive environment or the device itself. 

## 5. Conclusions

This study sought to investigate the effects of smart-speaker-survey technology on the panelist engagement and usability in an immersive consumer testing. As this is the first study to explore smart-speaker-survey technology in immersive sensory and consumer testing, a systematic approach was used. Dip consumers participated in three studies, each utilizing a different survey technology for product evaluation, and then answered a questionnaire on their engagement and the usability of the survey device. The environmental context of a cantina-style restaurant remained fixed throughout the experiment. The research team hypothesized that participants would find the smart-speaker surveys more engaging than a traditional tablet-based survey because a verbal experience is congruent with a restaurant environment, with the word-based Optimized smart-speaker survey being the most engaging. The reverse was found in that the Control survey was significantly more engaging, and differences were not observed between the two smart-speaker surveys. It was also hypothesized that the Control condition would score highest in usability, as all test participants were familiar with taking sensory and consumer questionnaires on computer-based platforms. This result was observed.

This research lays a foundation for the use of smart-speaker devices in sensory and consumer testing and provides ideas for future research involving smart-speaker surveys. Necessary modifications are required to improve the user experience during smart-speaker testing, including Alexa’s conversational capabilities and the device connectivity. Optimizing current smart-speaker surveys are critical for improving usability, the overall panelist experience, and the researcher’s execution experience. Without such enhancements, the use of smart-speaker surveys in immersive testing may not prove to be a worthwhile exercise. There is still potential for using these devices in traditional in-hall booth-testing environments, or for capturing in-the-moment product experiences at home, but sensory and consumer scientists need to understand the current technology constraints and appropriately design test questionnaires with them in mind. 

## Figures and Tables

**Table 1:** Commercial products and sample formulations for testing by product category.

Product Category	Product Name	Product Company	Mesquite Liquid Smoke Added per Product Container
**Guacamole**	Sabra Classic Guacamole with Lime (454 g container)	Sabra Dipping Co., LLC. White Plains, NY, USA	0 g No-smoke sample
	Simple Truth Traditional Guacamole (453 g container)	The Kroger Co. Cincinnati, OH, USA	11.3 g Low-smoke sample
	Simple Truth Traditional Guacamole (453 g container)	The Kroger Co. Cincinnati, OH, USA	22.7 g High-smoke sample
**Salsa**	La Mexicana Mild Salsa (453 g container)	Perimeter Brands LLC. Riviera Beach, FL, USA	0 g No-smoke sample
	Willy’s Original Mild Salsa (454 g container)	Willy’s Salsa Swanton, OH, USA	3.8 g Low-smoke sample
	Herdez Mild Salsa Casera (454 g container)	MegaMex Foods, LLC. Orange, CA, USA	45.4 g High-smoke sample
**Queso**	Tostitos Queso Blanco Dip Medium (425.2 g container)	Frito-Lay North America, Inc. Plano, TX, USA	0 g No-smoke sample
	Kroger Monterey Jack Queso Dip Medium (425 g container)	The Kroger Co. Cincinnati, OH, USA	7.1 g Low-smoke sample
	Kroger Monterey Jack Queso Dip Medium (425 g container)	The Kroger Co. Cincinnati, OH, USA	21.3 g High-smoke sample

**Figure A1:**
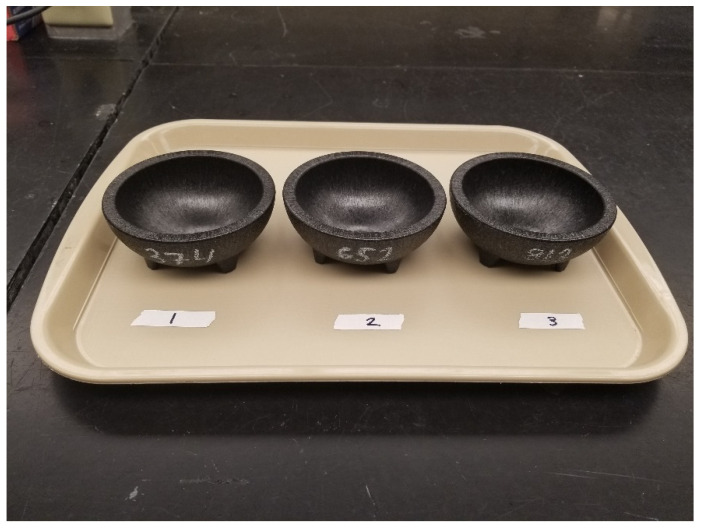
Example picture of molcajetes labeled with 3-digit codes and in proper serving order for testing. The serving order is designated by the 1, 2, and 3 stickers, with 1 being the first sample served, and 3 being the last.

## Data Availability

The data presented in this study are available upon request from the corresponding author.

## Appendix A. Product Preparation Protocol

### Appendix A.1. Night before Testing

- Label product containers with corresponding three-digit codes (and name if using GladWare);
- Weigh appropriate amount of Mesquite Liquid Smoke into 2 oz/59 mL plastic souffle cup (see table below);
- Pour/scoop product into plastic container with fitted lid (e.g., GladWare) for Simple Truth guacamoles and Herdez salsa. The other products have enough headspace for liquid to be added;
- Add liquid smoke to the product containers and stir until it is well incorporated;
- Place containers in refrigerator in sensory lab;
- Please note that the number of needed jars/containers of product will be determined based on the anticipated panel schedule/rotation plan for the following day.

**Table A1:** Product and liquid-smoke amounts for testing for product preparation.

Product Category	Product Name and Code	Mesquite Liquid Smoke to Add per Product Container
**Guacamole**	Sabra (201)	0 g—no-smoke sample
	Simple Truth (ST—Low), 453 g (196)	11.3 g—low-smoke sample
	Simple Truth (ST—High), 453 g (498)	22.7 g—high-smoke sample
**Salsa**	La Mexicana (La Mxana) (988)	0 g—no-smoke sample
	Willy’s (Low), 454 g (729)	3.8 g—low-smoke sample
	Herdez (High), 454 g (530)	45.4 g—high-smoke sample
**Queso**	Tostitos (652)	0 g—no-smoke sample
	Kroger (Low), 425 g (813)	7.1 g—low-smoke sample
	Kroger (High), 425 g (374)	21.3 g—high-smoke sample

### Appendix A.2. Morning of Testing

- Determine the number of times a sample will be evaluated for the day using the rotation plan and expected panel ID codes;
- Count out this number of pre-labeled 96 mL souffle cups and lids;
- Carefully match the labeled container in the refrigerator in 055 Howlett with the three-digit-code cup and fill with product until full (we will be serving approximately 89 mL of the sample at a time);
- Place lid on filled sample cup and put in refrigerator in Parker 055 back booth.

### Appendix A.3. During the Test Session

- Match the panel code and day of testing to determine the sample presentation order (in case this is not already labeled on a tray for you);
- Label molcajetes with three-digit codes and place in order on tray (see Figure A1 below) (note that if trays are already labeled, then they will have three-digit codes in place of 1, 2, 3);

- When the panelist is ready, take out the correct three-digit-code sample from the refrigerator;
- Find the corresponding molcajete with matching three-digit code and, using a plastic spoon, transfer the product from the souffle cup into the molcajete;
- Additional step for queso;
- Place the molcajete filled with the queso sample in the microwave in Parker 055, and heat for 30 s;
- Press 30 s button on microwave and let heat for 15 s;
- Open microwave and stir;
- Click start to resume heating and open microwave door when it reaches 1 s. This is to prevent participants from hearing the microwave go off (we found this an issue during pilot testing);
- Stir before serving;
- Throw away spoon and souffle cup while waiting to prepare the next sample;
- Repeat until the panelist has finished their set for the day;
- Clean space in between sessions and prepare for the next participant;
- Once all participants have finished for the day, clean the area, discard any leftover product in the fridge, and wash containers;
- Follow *Night Before Testing* preparation procedure to prepare product for the next day.

## Appendix B. Engagement Questionnaire and System Usability Scale Questions

**Table A2:** Engagement Questionnaire (EQ) statements grouped by engagement factor. Respondents rate their level of agreement to the following statements using a 7-point Likert scale (strongly disagree (1)–strongly agree (7)).

Factor	Item
Active Involvement	I lost interest in the task. *
	I was distracted. *
	I felt myself zoning out during the task. *
Purposeful Intent	I found the task meaningful.
	I felt dedicated to finish the task.
	I wanted to devote my full attention to the task.
	My contribution was significant to the outcome of the task.
Affective Value	I found the task captivating.
	During the task, I was enjoying myself.
	I was motivated to expend extra effort during the task.

* Reverse-coded item. Due to the negatively worded question format, values on the 7-point Likert scale are reversed (strongly disagree (7)–strongly agree (1)).

**Table A3:** System Usability Scale (SUS) statements grouped by user experience. All questions were presented to panelists in alternating fashion (i.e. positively framed item followed by negatively framed item). Respondents rate their level of agreement to the following statements using a 5-point Likert scale (strongly disagree (1)–strongly agree (5)).

Type of User Experience being Captured	Statement
Positive	I think that I would like to use this smart-speaker for sensory evaluation frequently.
	I thought the smart-speaker was easy to use.
	I found the various functions in the smart-speaker were well integrated.
	I would imagine most people would learn to use the smart-speaker very quickly.
	I felt very confident using the smart-speaker.
Negative	I found the smart-speaker unnecessarily complex.
	I think that I would need the support of a technical person to be able to use this smart-speaker.
	I thought that there was too much inconsistency in the smart-speaker.
	I found the smart-speaker very cumbersome (awkward) to use.
	I needed to learn a lot of things before I could get going with the smart-speaker.

## References

[1] Martini S. (2018). Editorial overview: Sensory science and consumer perception. Curr. Opin. Food Sci..

[2] Hannum M., Forzley S., Popper R., Simons C.T. (2019). Does environment matter? Assessments of wine in traditional booths compared to an immersive and actual wine bar. Food Qual. Prefer..

[3] Stelick A., Dando R. (2018). Thinking outside the booth—The eating environment, context and ecological validity in sensory and consumer research. Curr. Opin. Food Sci..

[4] Lawless H.T., Heymann H. (2010). Chapter 15—Consumer field tests and questionnaire design. Sensory Evaluation of Food: Principles and Practices.

[5] Bangcuyo R.G., Smith K.J., Zumach J.L., Pierce A.M., Guttman G.A., Simons C.T. (2015). The use of immersive technologies to improve consumer testing: The role of ecological validity, context and engagement in evaluating coffee. Food Qual. Prefer..

[6] Holthuysen N.T.E., Vrijhof M.N., de Wijk R.A., Kremer S. (2017). “Welcome on board”: Overall liking and just-about-right ratings of airplane meals in three different consumption contexts-laboratory, re-created airplane, and actual airplane. J. Sens. Stud..

[7] Man K., Patterson J.A., Simons C. (2023). The impact of personally relevant consumption contexts during product evaluations in virtual reality. Food Qual. Prefer..

[8] Young S.N. (2008). The neurobiology of human social behavior: An important but neglected topic. J. Psychiatry Neurosci..

[9] Purington A., Taft J.G., Sannon S., Bazarova N.N., Taylor S.H. “Alexa is my new BFF”: Social roles, user satisfaction, and personification of the amazon echo. Proceedings of the 2017 CHI Conference Extended Abstracts on Human Factors in Computing Systems.

[10] Kowalczuk P. (2018). Consumer acceptance of smart speakers: A mixed methods approach. J. Res. Interact. Mark..

[11] Lau J., Zimmerman B., Schaub F. (2018). Alexa, are you listening? Privacy perceptions, concerns and privacy-seeking behaviors with smart speakers. Proceedings of the ACM on Human-Computer Interaction, 2(CSCW).

[12] Sung E., Bae S., Han D.-I.D., Kwon O. (2021). Consumer engagement via interactive artificial intelligence and mixed reality. Int. J. Inf. Manag..

[13] Hannum M.E., Simons C.T. (2020). Development of the engagement questionnaire (EQ): A tool to measure panelist engagement during sensory and consumer evaluations. Food Qual. Prefer..

[14] Brooke J., Jordan P., Thomas B., Weerdmeester B. (1995). SUS: A ‘quick and dirty’ usability scale. Usability Evaluation in Industry.

[15] Lewis J.R., Sauro J. (2018). Item benchmarks for the system usability scale. J. Usability Stud..

[16] Sauro J., Lewis J.R., Sauro J., Lewis J.R. (2016). Chapter 8—Standardized usability questionnaires. Quantifying the User Experience.

[17] Hannum M.E., Forzley S., Popper R., Simons C.T. (2021). Application of the Engagement Questionnaire (EQ) to compare methodological differences in sensory and consumer testing. Food Res. Int..

[18] Kim I., Hopkinson A., van Hout D., Lee H. (2017). A novel two-step rating-based ‘double-faced applicability’ test. Part 1: Its performance in sample discrimination in comparison to simple one-step applicability rating. Food Qual. Prefer..

[19] Nicolas L., Marquilly C., O’mahony M. (2010). The 9-point hedonic scale: Are words and numbers compatible?. Food Qual. Prefer..

[20] Zwislocki J., Goodman D. (1980). Absolute scaling of sensory magnitudes: A validation. Percept. Psychophys..

[21] Price L. (2015). Responsibility: Identifying Purpose and Finding Meaning. Jurisprudence.

[22] Blicher A., Reinholdt-Dunne M.L., Hvenegaard M., Winding C., Petersen A., Vangkilde S. (2020). Engagement and disengagement components of attentional bias to emotional stimuli in anxiety and depression. J. Exp. Psychopathol..

[23] Georgiou G., Bleakley C., Hayward J., Russo R., Dutton K., Eltiti S., Fox E. (2005). Focusing on fear: Attentional disengagement from emotional faces. Vis. Cogn..

[24] Larson R.B. (2019). Controlling social desirability bias. Int. J. Mark. Res..

[25] McCambridge J., de Bruin M., Witton J. (2012). The Effects of Demand Characteristics on Research Participant Behaviours in Non-Laboratory Settings: A Systematic Review. PLoS ONE.

[26] Baethge A., Vahle-Hinz T., Schulte-Braucks J., Van Dick R. (2017). A matter of time? Challenging and hindering effects of time pressure on work engagement. Work. Stress.

[27] Kirsh D. (2000). A few thoughts on cognitive overload. Intellectica.

[28] Speier C., Valacich J.S., Vessey I. (1999). The influence of task interruption on individual decision making: An information overload perspective. Decis. Sci..

[29] Wang S., Lilienfeld S.O., Rochat P. (2015). The uncanny valley: Existence and explanations. Rev. Gen. Psychol..

[30] Packard G., Berger J. (2021). How concrete language shapes customer satisfaction. J. Consum. Res..

[31] Amazon (2021). Welcome to Amazon Sidewalk. https://www.amazon.com/Amazon-Sidewalk/b?ie=UTF8&node=21328123011.

[32] Tempelaar D., Rienties B., Nguyen Q. (2020). Subjective data, objective data and the role of bias in predictive modelling: Lessons from a dispositional learning analytics application. PLoS ONE.

